# Supplementary material for: Mycobacterial DnaB helicase intein as oxidative stress sensor
Source: Nat Commun. 2018 Oct 19;9:4363. doi: 10.1038/s41467-018-06554-x (PMC6195587; doi:10.1038/s41467-018-06554-x)
Supplement: Supplementary file 1 — Supplementary Information [file 41467_2018_6554_MOESM1_ESM.pdf]

Mycobacterial DnaB helicase intein as oxidative stress sensor

D. S. Kelley, C. W. Lennon et al.

SUPPLEMENTARY INFORMATION

FIGURES

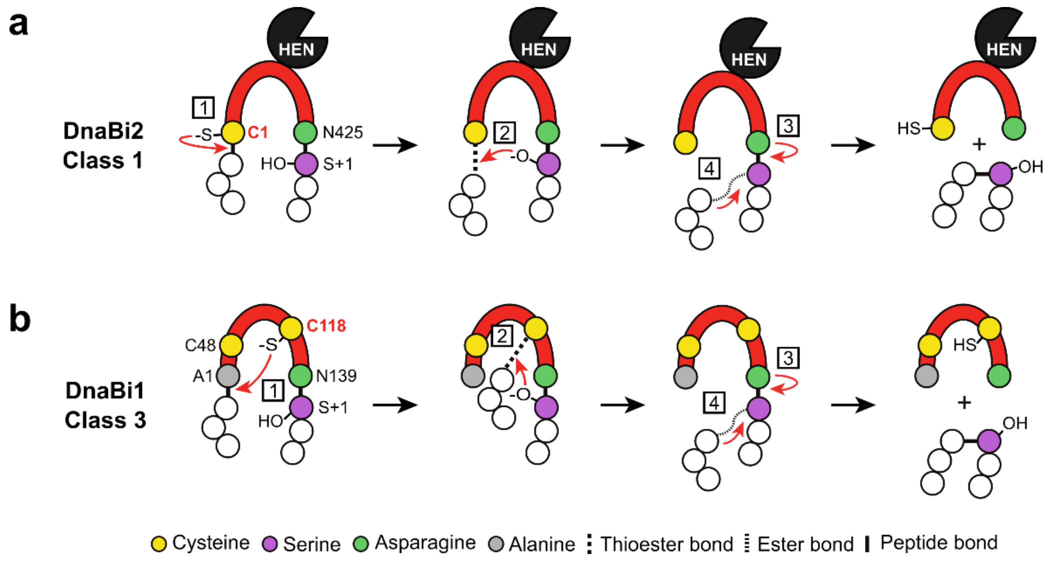

Supplementary Figure 1. Expanded splicing mechanisms for class 1 and class 3 inteins

a. Class 1 intein splicing mechanism. Cys1 attacks the preceding amide bond forming a thioester (step 1). Ser+1 then performs a second nucleophilic attack (step 2). This branched intermediate is resolved by terminal Asn cyclization (step 3) and, finally, there is an O-N acyl rearrangement to form the native peptide link (step 4).

b. Class 3 intein splicing mechanism. Internal Cys118 attacks the amide bond at the N-extein-intein junction (step 1). This is followed by a second nucleophilic attack by Ser+1 (step 2) and, in analogy to class 1 inteins, cyclization by the terminal Asn (step 3) to free the intein. Step 4 is the reformation of the native peptide bond, as described for class 1.

Residue numbers based on *Msm* DnaB inteins.

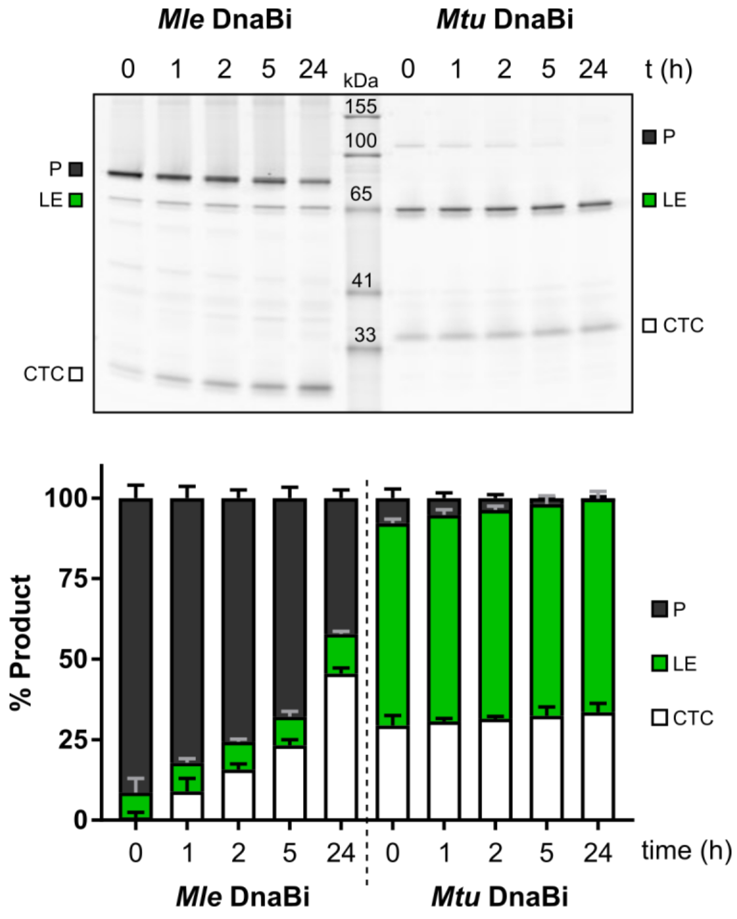

Supplementary Figure 2. Splicing of MIG *Mle* and *Mtu* DnaB inteins

The MIG *Mle* (left) and *Mtu* (right) DnaB inteins display similar splicing characteristics to their *Msm* counterparts, where MIG *Mle* DnaBi splices slowly, like *Msm* DnaBi1, and MIG *Mtu* DnaBi splices quickly, like *Msm* DnaBi2. Samples are run under non-reducing conditions with loading dye lacking β-mercaptoethanol. The size difference between the C-terminal cleavage (CTC) products is attributable to distinct C-extein residue composition between *Mle* DnaBi and *Mtu* DnaBi, resulting in charge variances which can cause spurious migration. The ratio of splice products was quantified and plotted (bottom). Data are representative of three biological replicates and values are expressed as mean ± s.d.

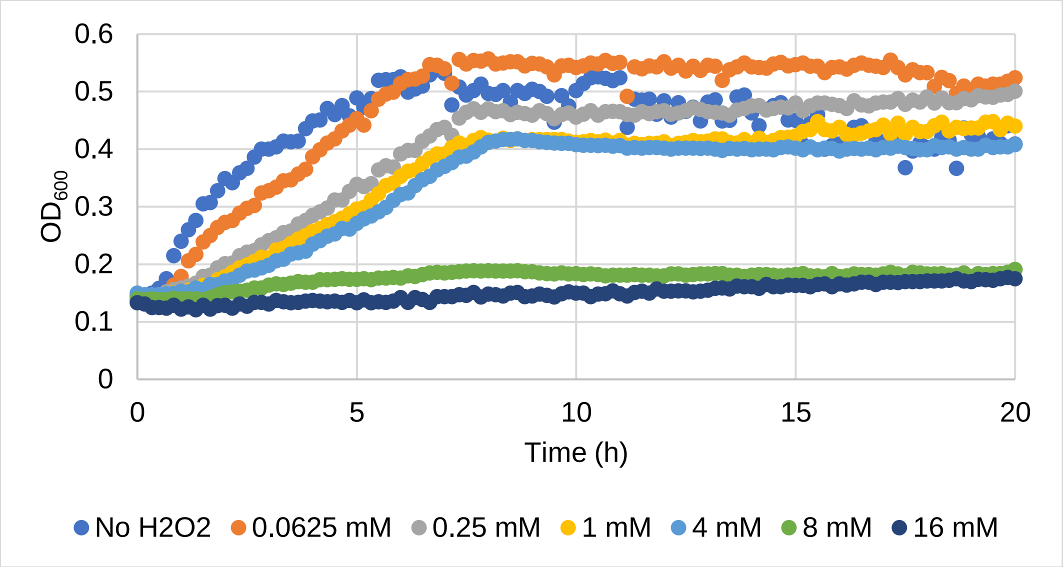

Supplementary Figure 3. Growth curve of *M. smegmatis* under H_2O_2 stress

M. smegmatis cells were diluted to a starting OD_{600} of ~0.15 and H_2O_2 was either not added or added at the indicated concentrations. Cells were grown at 37°C with shaking in a microplate reader (Biotek) and OD_{600} readings taken every 10 minutes. Growth inhibition occurred in a concentration-dependent manner, with killing at the highest H_2O_2 concentrations (8 and 16 mM). Data is representative of biological replicates (n=3) performed under similar conditions.

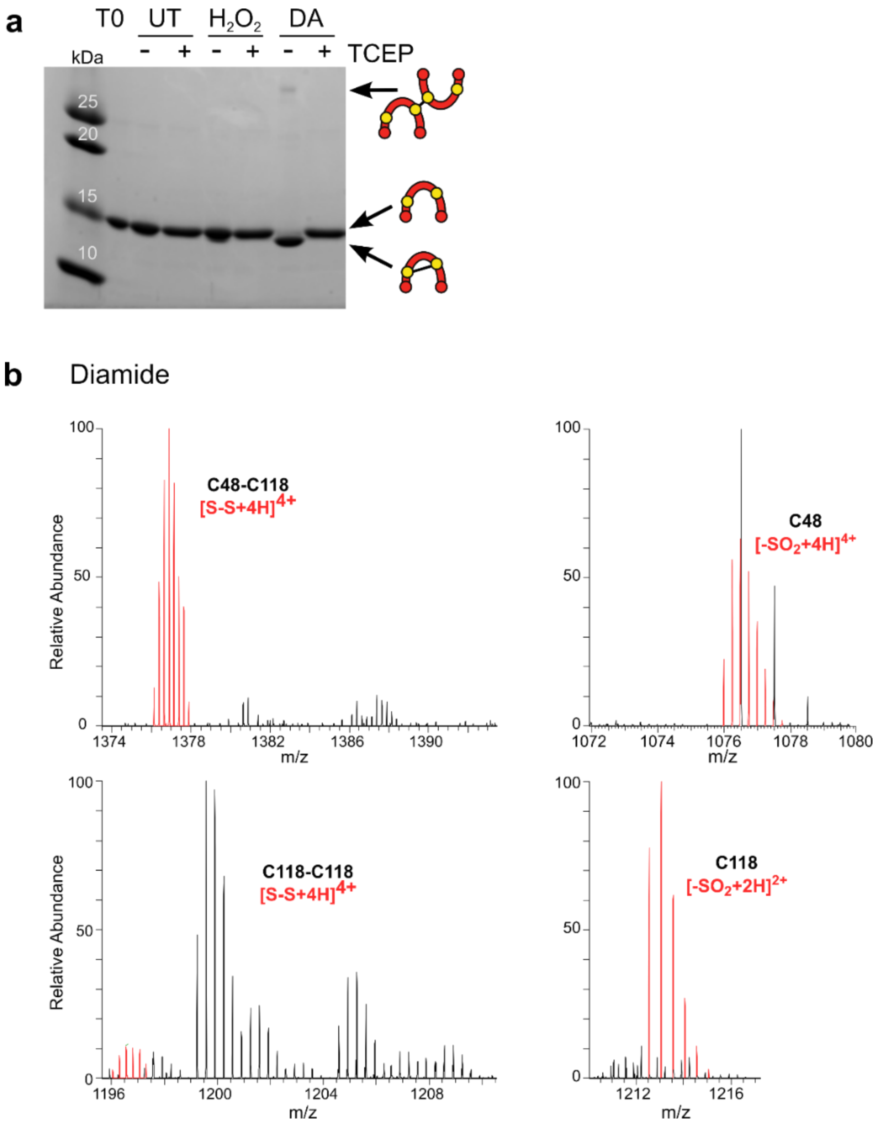

Supplementary Figure 4. Additional ROS-induced modifications of DnaBi1

a. DnaBi1 ROS-induced modifications are reversible. Purified DnaBi1 at 10 µM was treated with 1 mM ROS reagents (H_2O_2; DA, diamide) or buffer (UT, untreated) anaerobically. After a 15-min incubation at 30°C, samples were either put on ice or treated with 40 mM tris(2-carboxyethyl)phosphine (TCEP) on ice and separated on an SDS-PAGE gel under non-reducing conditions in the anaerobic chamber. Gel is representative of three technical replicates.

b. Diamide-induced modifications of DnaBi1. MS revealed that diamide caused both Cys48-Cys118 intra- (top left) and Cys118-Cys118 inter- (bottom left) disulfide bonds. Additionally, irreversible sulfinic acid modifications were observed on both Cys48 (top right) and Cys118 (bottom right). Peaks of interest are indicated in red.

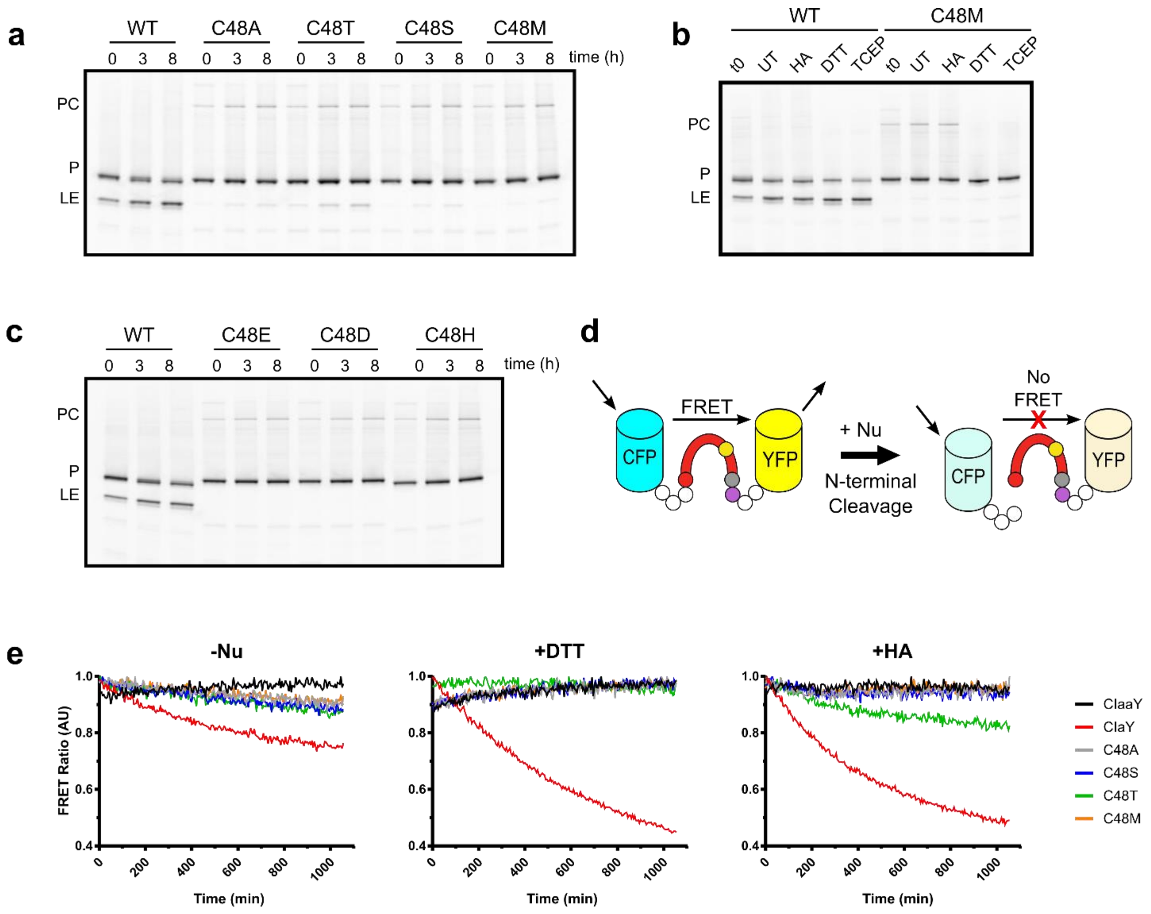

Supplementary Figure 5. Cys48 mutants are splicing defective and accumulate intermolecular disulfide-bonded precursor

a. Cys48 MIG mutants are splicing impaired. The splicing of MIG DnaBi1 WT and Cys48 mutants to Ala, Ser, Thr, and Met were compared. All four mutants showed a diminished ability to splice and, to various degrees, accumulated upper molecular weight precursor conformers (PC). C48T was the most active mutant, resulting in the most LE. Gel is representative of three biological replicates.

b. High molecular weight bands are likely intermolecular disulfide-bonded precursors. These upper bands could either be precursor-precursor disulfide bonded species, susceptible to reduction, or stalled branched intermediates, which could be cleaved by a nucleophile. To determine the identity of this high molecular weight product, termed precursor conformer (PC), a 5-h time-point for both WT and C48M was treated with 100 mM hydroxylamine (HA), an external nucleophile, 20 mM dithiothreitol (DTT), a nucleophile and reducing agent, or 20 mM

TCEP, a reducing agent, for an additional 3 hours at 30°C. Addition of TCEP or DTT, but not HA, caused the PC bands to resolve. This implies that the PC bands are a reversible product, implicating intermolecular disulfide-bonded precursors rather than stalled intermediate products. Gel is representative of three biological replicates.

c. Additional MIG DnaBi1 mutants are also splicing inactive. Three additional Cys48 mutants, C48H, C48D, and C48E, were made in the MIG DnaBi1 to address the potential nucleophilicity of Cys48 on splicing. All three mutants were unable to splice and accumulated PC over time. Gel is representative of three biological replicates.

d. Overview of the FRET assay. DnaBi1 was cloned between CFP and YFP, generating a fusion protein of CFP-intein-YFP (CIY)[1]. When the intein is present, FRET between the two fluorophores can occur (left). A terminal Asn to Ala mutation (gray circle) prevents the intein from undergoing complete splicing. This allows the loss of FRET (right) to serve as a proxy for the first step of splicing, or N-terminal cleavage, in the presence of an external nucleophile (Nu), such as DTT or HA.

e. Cys48 mutants are defective in N-terminal cleavage. To test the ability of these mutants to initiate splicing, the same mutations as in panel A were made in a FRET assay construct (see panel d). The DnaBi1 parental construct (CIaY), was used as the template to make all subsequent mutations. An N-terminal cleavage (NTC)-inactive version was also constructed (CIaaY) by making an additional Cys118Ala mutation. In the absence of an external nucleophile (left), CIaY can undergo some NTC but this is bolstered in the presence of DTT or HA. However, none of the mutants can undergo DTT-induced NTC (middle) and only C48T can undergo minimal HA-induced NTC (right). Plots are the average of two technical replicates.

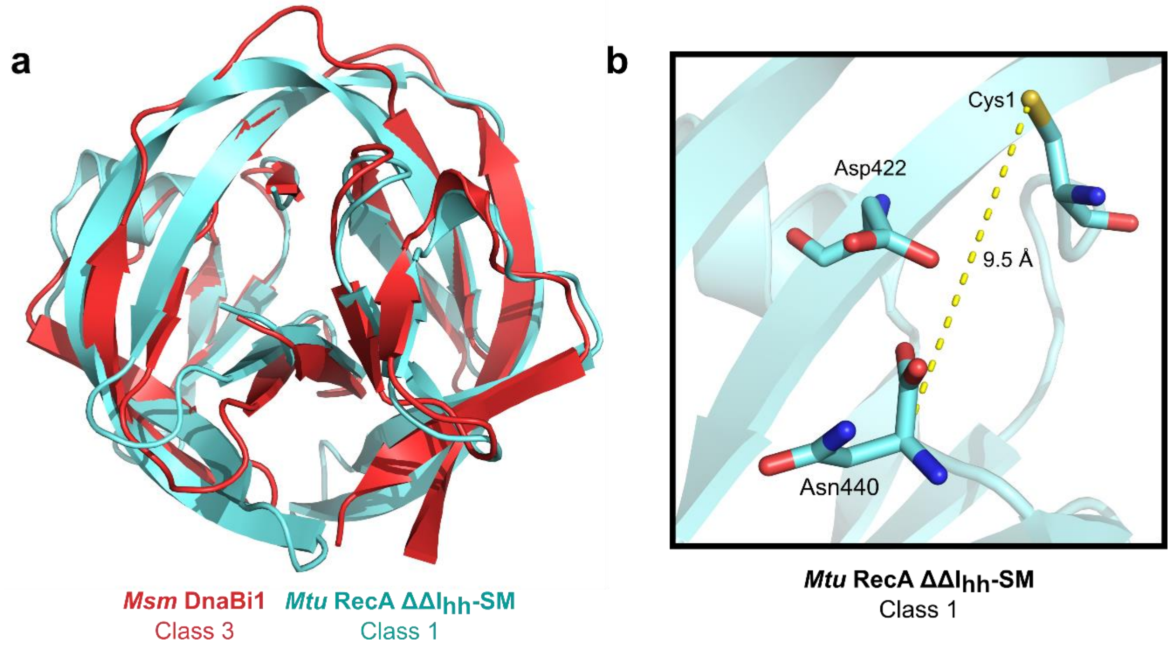

Supplementary Figure 6. Structural comparison of class 3 and class 1 inteins

a. Alignment of class 3 DnaBi1 and class 1 *Mtu* RecAi. The *Msm* DnaBi1 class 3 intein (red) was aligned to the *Mtu* RecA $\Delta\Delta I_{hh}$-SM class 1 structure (PDB 2IN0)[2] in cyan by 3D-BLAST (RMSD = 2.12; http://3d-blast.life.nctu.edu.tw)[3].

b. RecAi has a larger distance between the initiating nucleophile and C-extein junction. The distance between the initiating nucleophile, Cys1, and the C-extein junction for RecAi is approximately 9.5 Å, on par with other class 1 inteins[4]. However, Cys118 is centrally located between both the N- and C-extein junctions, with shorter distances for either cysteine conformation (see Figure 6e).

Supplementary Figure 7. Uncropped gels and blots

Dashed red boxes indicate cropped regions.

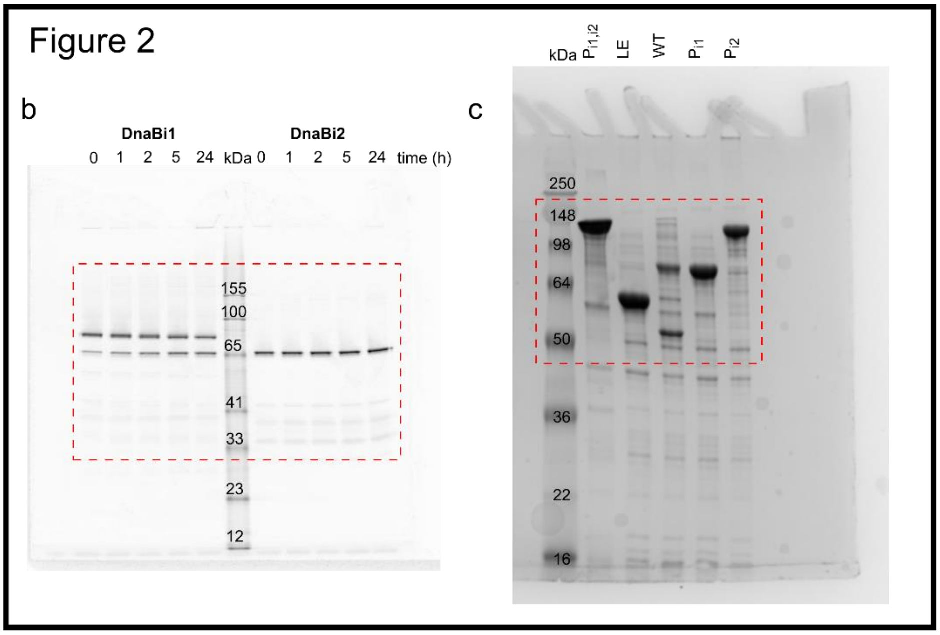

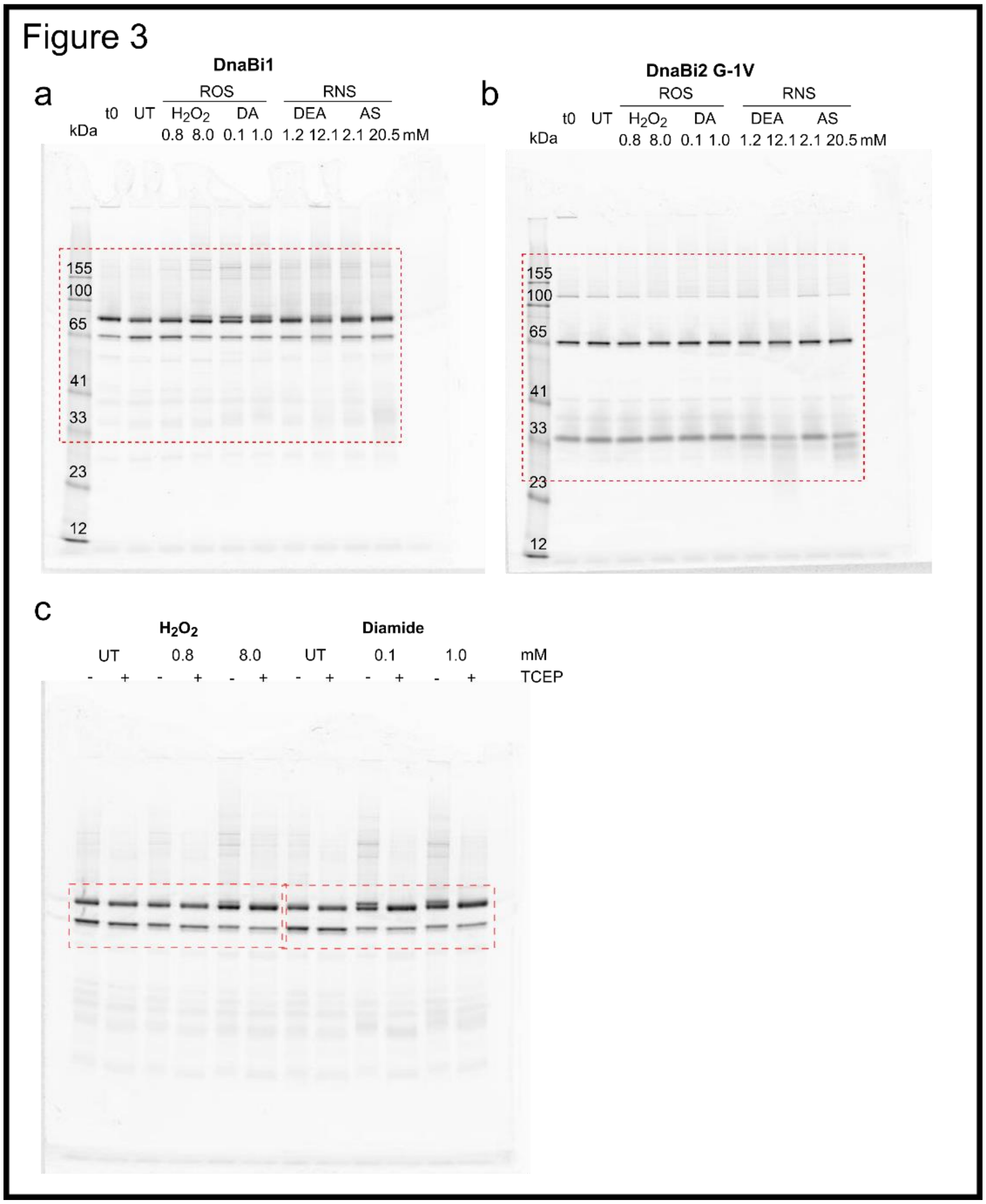

Figure 4

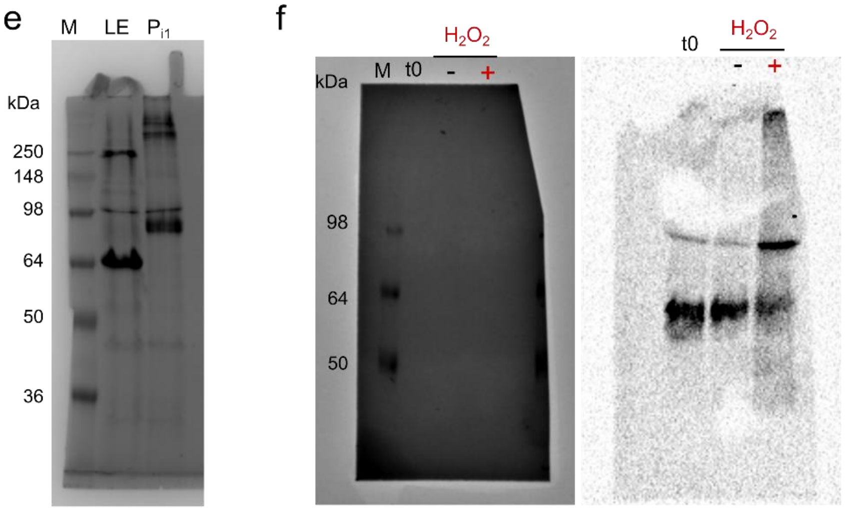

Figure 5a

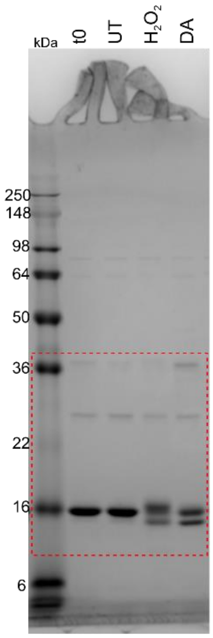

Supplementary Figure 2

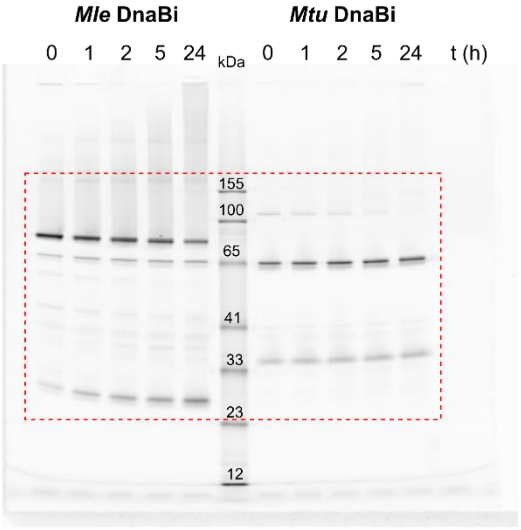

Supplementary Figure 4

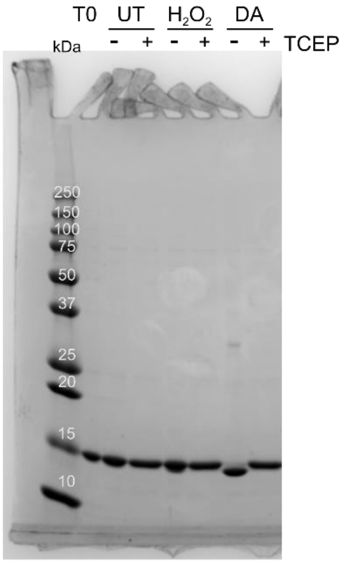

Supplementary Figure 5

a

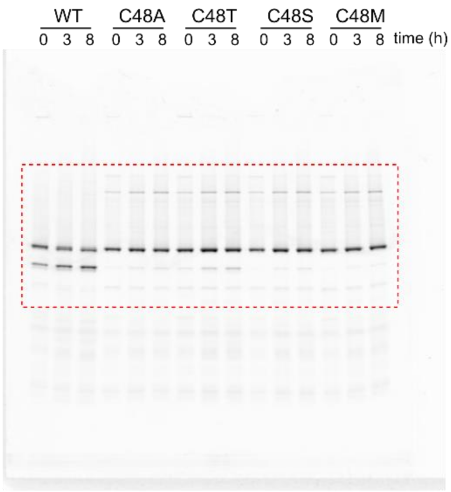

b

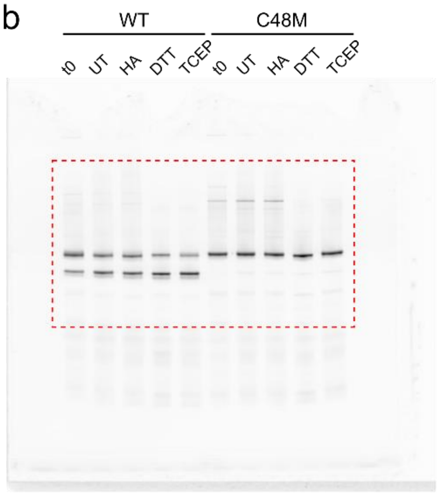

c

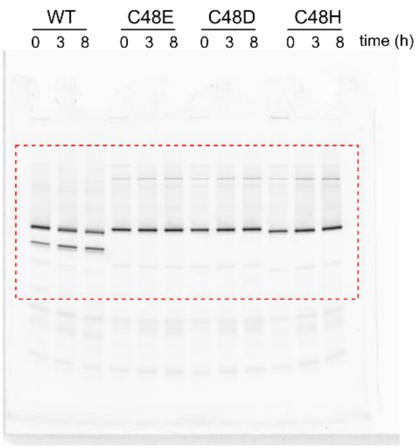

TABLES

Supplementary Table 1. Cys118 B factor values

	Chain A	Chain B	Chain C
B factor	51.17 Å^2	conformation a; 29.66 Å^2	54.90 Å^2
		conformation b; 41.34 Å^2	

Supplementary Table 2. Bacterial strains and constructs

Strain/Plasmid	Features and Comments	Source
E. coli Strains		
DH5α	F⁻ *endAI recAl hsdRl7* (rK⁻ mK⁻) *deoR supE44 thi-J gyrA96 relA*	Gibco-BRL
MG1655(DE3)	F⁻ (λDE3) *ilvG rfb50 rph*1	James Imlay
BL21(DE3)	F⁻ *ompT hsd*S$_B$(r_B^- m_B^-) *gal dcm* (λDE3)	Novagen
JM109	*end*A1, *rec*A1, *gyr*A96, *thi*, *hsd*R17 (r_k^-, m_k^+), *rel*A1, *sup*E44, Δ(*lac*-*pro*AB), [F´ *tra*D36, *pro*AB, *laq*I^{q}ZΔM15]	Stratagene
MC1061	*hsdR mcrB araD139* Δ(*araABD-leu*)*7697* Δ*lacX74 galU galK rpsL thi relA1 spoT1*	[1]
B834(DE3)	F⁻ *ompT hsdS*$_B$(r_B^- m_B^-) *gal dcm met* (λDE3)	Novagen
Plasmids		
pACYCDuet-1	Expression vector, T7 promoter, Cam^R.	Novagen
pACYC MIG SufB	Used as cloning backbone by removing *M. tuberculosis* SufB intein insert using ClaI/SphI.	[5]
pACYC MIG DnaBi1	*M. smegmatis* DnaB intein 1 flanked by short native exteins (N-Extein: AARPGVGK; C-Extein: STLGLDFMRS) cloned into ClaI/SphI sites between MBP and GFP coding sequences in pACYCDuet-1 backbone.	Present Study
pACYC MIG DnaBi2	*M. smegmatis* DnaB intein 2 flanked by short native exteins (N-Extein: PQVSDLRESG; C-Extein: SLEQDADM) cloned into ClaI/SphI sites between MBP and GFP coding sequences in pACYCDuet-1 backbone.	Present Study
pACYC MIG DnaBi2 G-1V	Same as pACYC MIG DnaBi2 with a G-1V mutation in the N-extein.	Present Study
pACYC MIG *Mle* DnaBi	*M. leprae* DnaB intein flanked by short native exteins (N-Extein: AARPGVGK; C-Extein: SNTLGLDFMRS) cloned into ClaI/SphI sites between MBP and GFP coding sequences in pACYCDuet-1 backbone.	Present Study
pACYC MIG *Mtu* DnaBi	*M. tuberculosis* DnaB intein flanked by short native exteins (N-Extein: PMLADLRESG; C-Extein: SLEQDAD) cloned into ClaI/SphI sites between MBP and GFP coding sequences in pACYCDuet-1 backbone.	[5]
pACYC MIG DnaBi1 C48A/S/T/M/H/D/E	Same as pACYC MIG DnaBi1 with the indicated amino acid substitution at Cys48.	Present Study
pXI	IPTG-inducible expression vector, chitin binding domain for purification, Amp^R.	[6]

pXI DnaBi1	*M. smegmatis* DnaB intein 1 with short native N-extein (GVGK) in pXI backbone at EcoRI/HindIII sites.	Present Study
pET47b	Expression vector, T7 promoter, N-terminal His_6 tag, Precission cleavage, Kan^R.	Novagen
pET47b DnaB WT	*M. smegmatis* full-length DnaB with splicing active inteins in pET47b backbone at EcoRI/XhoI sites.	Present Study
pET47b DnaB $P_{i1,i2}$	*M. smegmatis* full-length DnaB with mutations C118A/N139A in intein 1 and C1A/N425A in intein 2 to inhibit protein splicing. Mimics precursor protein with both inteins present.	Present Study
pET47b DnaB P_{i1}	*M. smegmatis* full-length *dnaB* gene with mutations C118A/N139A in intein 1 to inhibit splicing and intein 2 removed by SOEing PCR. Mimics alternative precursor protein with intein 1 still present.	Present Study
pET47b DnaB P_{i2}	*M. smegmatis* full-length *dnaB* gene with mutations C1A/N425A in intein 2 to inhibit splicing and intein 1 removed by SOEing PCR. Mimics alternative precursor protein with intein 2 still present.	Present Study
pET47b DnaB LE	*M. smegmatis* full-length *dnaB* gene with both inteins removed. Mimics functional DnaB protein with both inteins having spliced.	Present Study
pMBC283	Mycobacterial shuttle vector.	Kathleen McDonough
pMBC283 Kan^R-	Mycobacterial shuttle vector with Kan^R. Splice or Die construct.	Present Study
pMBC283 Kan^R-DnaBi1 WT	Mycobacterial shuttle vector with Kan^R interrupted by *Msm* DnaBi1 wild-type at Ser154. Splice or Die construct.	Present Study
pMBC283 Kan^R-DnaBi1 C118A	Mycobacterial shuttle vector with Kan^R interrupted by *Msm* DnaBi1 C118A at Ser154. Splice or Die construct.	Present Study
C-I-Y DnaE	Original FRET assay construct with *Synechocystis* sp PCC6803 DnaE intein. Used as template for CIY DnaBi1 cloning.	[1]
CIY DnaBi1 WT (CIY)	DnaBi1 cloned between CFP and YFP with short native exteins (N-extein, PGVGK ; C-extein, STLGL) in pBAD33 backbone at XhoI/PstI sites.	Present Study
CIY DnaBi1 NA (CIaY)	Same as CIY with Asn139 to Ala mutation.	Present Study
CIY DnaBi1 AA (CIaaY)	Same as CIaY with Cys118 to Ala mutation.	Present Study
CIY DnaBi1 NA C48A/S/T/M	Same as CIaY with the indicated amino acid mutation at Cys48.	Present Study

Supplementary Table 3. Oligonucleotides

Oligo ID	Sequence (5′ to 3′)	Application
IDT3535	ggggcatgcagctcgtccgggtgtgggcaaggcg	*Mle* DnaBi forward primer for MIG with SphI site.
IDT3536	cccatcgatcgaccgcatgaagtccagcccaagggtcga	*Mle* DnaBi reverse primer for MIG with ClaI site.
IDT3683	atgtcggcgtcctgctccaggctccccgactcacgaagatcgg	Reverse SOEing with complementation to extein 2 and overlap with extein 3.
IDT3684	tccgatcttcgtgagtcggggagcctggagcaggacgccga	Forward SOEing with complementation to extein 3 and overlap with extein 2.
IDT3685	gcatgaagtccagcccgagtgtcgacttacccacaccgggacgc	Reverse SOEing with complementation to extein 1 and overlap with extein 2.
IDT3686	gcgtcccggtgtgggtaagtcgacactcgggctggacttc	Forward SOEing with complementation to extein 2 and overlap with extein 1.
IDT3941	tcgagggaaggccttacatgcatgctgcgcgtcccggtgtgggt	*Msm* DnaBi1 forward primer for MIG with InFusion SphI end.
IDT3942	cttctcctttgctcatatcgatcgaccgcatgaagtccagcc	*Msm* DnaBi1 reverse primer for MIG with InFusion ClaI end.
IDT3943	tcgagggaaggccttacatgcatgctccgcaggtgtccgatctt	*Msm* DnaBi2 forward primer for MIG with InFusion SphI end.
IDT3944	cttctcctttgctcatatcgatcatgtcggcgtcctgctc	*Msm* DnaBi2 reverse primer for MIG with InFusion ClaI end.
IDT4285	ttgtggcagcttcaagaattcggtgtgggtaaggcgctc	*Msm* DnaBi1 forward primer for pXI with EcoRI InFusion ends.
IDT4286	aacgacggccagtgccaagcttttagttgtgcgtgggaaccatgc	*Msm* DnaBi1 reverse primer for pXI with HindIII InFusion ends.
IDT4301	ccgatcttcgtgagtcgnnstgcatgaccgcgaacacccgtatcttgc	*Msm* DnaBi2 G-1 random mutagenesis sense primer. N = any nucleotide, S = G or C.
IDT4302	gcaagatacgggtgttcgcggtcatgcasnncgactcacgaagatcgg	*Msm* DnaBi2 G-1 random mutagenesis anti-sense primer. N = any nucleotide, S = G or C.

IDT4310	acaaaggttccggtggctcgagacccggtgtgggtaaggcg	*Msm* DnaBi1 forward primer for amplification and cloning into pBAD33 CIY with XhoI InFusion ends.
IDT4311	gacatagagccaccggaaccctgcagcagcccgagtgtcgagtt	*Msm* DnaBi1 reverse primer for amplification and cloning into pBAD33 CIY with PstI InFusion ends.
IDT4569	aggttaattaagcctcgagtcagcgagccatattcgtg	*Msm dnaB* reverse primer for amplification and cloning into pET47b with XhoI InFusion ends.
IDT4900	ggtaccaggatccgaattcattggcagtcgtggacgac	*Msm dnaB* forward primer for amplification and cloning into pET47b with EcoRI InFusion ends.
IDT5074	cgtgcccgtgcgggctgtcgaggtggac	*Msm* DnaBi1 mutagenic primer to mutate Cys118 to Ala.
IDT5075	tggttcccacgcacgcctcgacactcgggc	*Msm* DnaBi1 mutagenic primer to mutate Asn139 to Ala.
IDT5076	cttcgtgagtcgggggccatgaccgcgaacac	*Msm* DnaBi2 mutagenic primer to mutate Cys1 to Ala.
IDT5077	gcatctcagcgcacgccagcctggagcagg	*Msm* DnaBi2 mutagenic primer to mutate Asn425 to Ala.
IDT5295	gatgctcgggcggcccgcctacgtcgtcgagttc	*Msm* DnaBi1 Cys48 to Ala mutagenic anti-sense primer.
IDT5296	gaactcgacgacgtaggcgggccgcccgagcatc	*Msm* DnaBi1 Cys48 to Ala mutagenic anti-sense primer.
IDT5358	tgctcgggcggcccagctacgtcgtcgag	*Msm* DnaBi1 Cys48 to Ser mutagenic sense primer.
IDT5359	ctcgacgacgtagctgggccgcccgagca	*Msm* DnaBi1 Cys48 to Ser mutagenic anti-sense primer.
IDT5360	gtgatgctcgggcggcccatgtacgtcgtcgagttctcc	*Msm* DnaBi1 Cys48 to Met mutagenic sense primer.
IDT5361	ggagaactcgacgacgtacatgggccgcccgagcatcac	*Msm* DnaBi1 Cys48 to Met mutagenic anti-sense primer.

IDT5362	gatgctcgggcggcccacctacgtcgtcgagttc	*Msm* DnaBi1 Cys48 to Thr mutagenic sense primer.
IDT5363	gaactcgacgacgtaggtgggccgcccgagcatc	*Msm* DnaBi1 Cys48 to Thr mutagenic anti-sense primer.
IDT5787	gaactcgacgacgtagtggggccgcccgagcatc	*Msm* DnaBi1 Cys48 to His mutagenic anti-sense primer.
IDT5788	gatgctcgggcggccccactacgtcgtcgagttc	*Msm* DnaBi1 Cys48 to His mutagenic sense primer.
IDT5789	gaactcgacgacgtagtcgggccgcccgagcatc	*Msm* DnaBi1 Cys48 to Asp mutagenic anti-sense primer.
IDT5790	gatgctcgggcggcccgactacgtcgtcgagttc	*Msm* DnaBi1 Cys48 to Asp mutagenic sense primer.
IDT5791	ggagaactcgacgacgtactcgggccgcccgagcatcac	*Msm* DnaBi1 Cys48 to Glu mutagenic anti-sense primer.
IDT5792	gtgatgctcgggcggcccgagtacgtcgtcgagttctcc	*Msm* DnaBi1 Cys48 to Glu mutagenic sense primer.
IDT5807	attgaaaaaggaagagtatgagtattcaacatttccgtgtcgccc	pMBC283 forward inverse primer for NEBuilder
IDT5808	gcgaaacgatcctcatcctg	pMBC283 reverse inverse primer for NEBuilder.
IDT5809	caggatgaggatcgtttcgcatgagccatattcaacgg	Amplification of Kan^R cassette forward primer.
IDT5810	catactcttcctttttcaatttagaaaaactcatcgagc	Amplification of Kan^R cassette reverse primer.

Supplementary Table 4. MolProbity analysis of the disulfide-linked DnaBi1 intein model

Clashscore, all atoms	3.73
Favored rotamers	82.3%
Ramachandran favored	87.1%
MolProbity score	2.43
Imperfect bonds	0.5%
Imperfect angles	2.2%
Cis peptides	0.0%
Twisted peptides	2.7%

SUPPLEMENTARY REFERENCES

1 Amitai, G., Callahan, B. P., Stanger, M. J., Belfort, G. & Belfort, M. Modulation of intein activity by its neighboring extein substrates. *Proc. Natl. Acad. Sci. U. S. A.* **106**, 11005-11010 (2009).

2 Van Roey, P. *et al.* Crystallographic and mutational studies of *Mycobacterium tuberculosis* recA mini-inteins suggest a pivotal role for a highly conserved aspartate residue. *J. Mol. Biol.* **367**, 162-173 (2007).

3 Tung, C. H., Huang, J. W. & Yang, J. M. Kappa-alpha plot derived structural alphabet and BLOSUM-like substitution matrix for rapid search of protein structure database. *Genome Biol.* **8**, R31(2007).

4 Eryilmaz, E., Shah, N. H., Muir, T. W. & Cowburn, D. Structural and dynamical features of inteins and implications on protein splicing. *J. Biol. Chem.* **289**, 14506-1451 (2014).

5 Topilina, N. I. *et al.* SufB intein of *Mycobacterium tuberculosis* as a sensor for oxidative and nitrosative stresses. *Proc. Natl. Acad. Sci. U. S. A.* **112**, 10348-10353 (2015).

6 Hiraga, K., Derbyshire, V., Dansereau, J. T., Van Roey, P. & Belfort, M. Minimization and stabilization of the *Mycobacterium tuberculosis* recA intein. *J. Mol. Biol.* **354**, 916-926 (2005).
